# Association of body roundness index with female infertility: 2013–2018 NHANES

**DOI:** 10.3389/fnut.2024.1416637

**Published:** 2024-10-30

**Authors:** Wenhui Wang, Shengdi Hou, Kun Wang, Bin Ling, Huan Yu

**Affiliations:** ^1^Department of Gynecologic Oncology, Beijing Obstetrics and Gynecology Hospital, Capital Medical University, Beijing Maternal and Child Health Care Hospital, Beijing, China; ^2^Department of Obstetrics and Gynecology, China-Japan Friendship Hospital, Beijing, China; ^3^Graduate School, Capital Medical University, Beijing, China

**Keywords:** BRI, infertility, cross-sectional study, NHANES, women health

## Abstract

**Aim:**

This study aims to understand the association between body roundness index (BRI) and female infertility prevalence. Infertility is a public health concern with significant implications for individuals’ well-being and rights.

**Methods:**

All individuals who completed the National Health and Nutrition Examination Survey (NHANES) between 2013 and 2018 were initially included in this cross-sectional study. Following the screening, 2,777 eligible participants were selected for analysis from the original pool of 10,375 participants. Trained operators conducted anthropometric measurements, including height, weight, and waist circumference. The BRI was then calculated based on established research. Data from infertility status questionnaires were gathered from the NHANES database for all participants, with self-reported infertility serving as the study outcome. Multivariable logistic regression and restricted cubic splines (RCS) were employed to investigate the relationship between BRI and infertility. Subgroup analyses were also conducted to further explore the association between BRI and infertility.

**Results:**

Upon analyzing the baseline characteristics of all women in the study, notable distinctions were identified in the clinical and demographic features between fertile and infertile women. Our investigation revealed a positive correlation between BRI and the likelihood of infertility in both weighted and unweighted multiple logistic regression models. Additionally, BRI exhibited a significant association with infertility in both continuous and categorical forms. Utilizing RCS curves, we noted a linear escalation in the prevalence of infertility with rising BRI values. Subgroup analyses provided further clarity on these observations.

**Conclusion:**

Our study demonstrates a statistically significant positive correlation between BRI and the prevalence of infertility across diverse populations, suggesting potential implications for infertility prevention and treatment. Future prospective cohort studies will explore this association and understand the underlying mechanisms.

## Introduction

Infertility is a global health concern with significant implications for individuals’ well-being and rights (1–3). Around 48.5 million people worldwide are affected by infertility, leading to psychological, social, and reproductive health challenges, especially in underdeveloped regions (4). The condition is influenced by genetic, endocrine, and environmental factors (5). Hence, the prevention and management of infertility are imperative for the overall health and psychological welfare of women.

Abdominal obesity, characterized by fat accumulation around visceral organs in the abdominal cavity, is a prevalent issue linked to infertility (6, 7). Studies have shown a strong association between abdominal obesity and conditions like ovulation disorders, Polycystic Ovary Syndrome (PCOS), insulin resistance, abnormal estrogen levels, and disruptions in the reproductive cycle (8–12). Body mass index (BMI) is often used to assess obesity, still it may not accurately predict infertility prevalence due to its limitations in distinguishing between fat and muscle mass, and not considering fat distribution, particularly in cases of abdominal obesity (11).

In recent years, the body roundness index (BRI) has been recognized as a new anthropometric index that combines height and waist circumference measurements to provide a detailed picture of body shape and fat distribution (13). Higher BRI values indicate more abdominal fat, which can lead to health issues. Studies have linked high BRI levels to metabolic syndrome, fatty liver, cardiovascular disease, and psychological distress, providing a new tool for health assessment (14–20). Nevertheless, the relationship between BRI and infertility remains uncertain.

Further research on the relationship between BRI and infertility issues, along with an exploration of the physiological and pathological mechanisms involved, is crucial for enhancing the prevention and treatment of infertility and advancing reproductive health. In this study, we utilized data from the National Health and Nutrition Examination Survey (NHANES) to conduct a significant cross-sectional study, for the first time, examining the link between BRI and infertility among women in the United States. The results of this study could provide valuable insights into the prevention and treatment of infertility.

## Methods

### Study population

**Figure 1:**
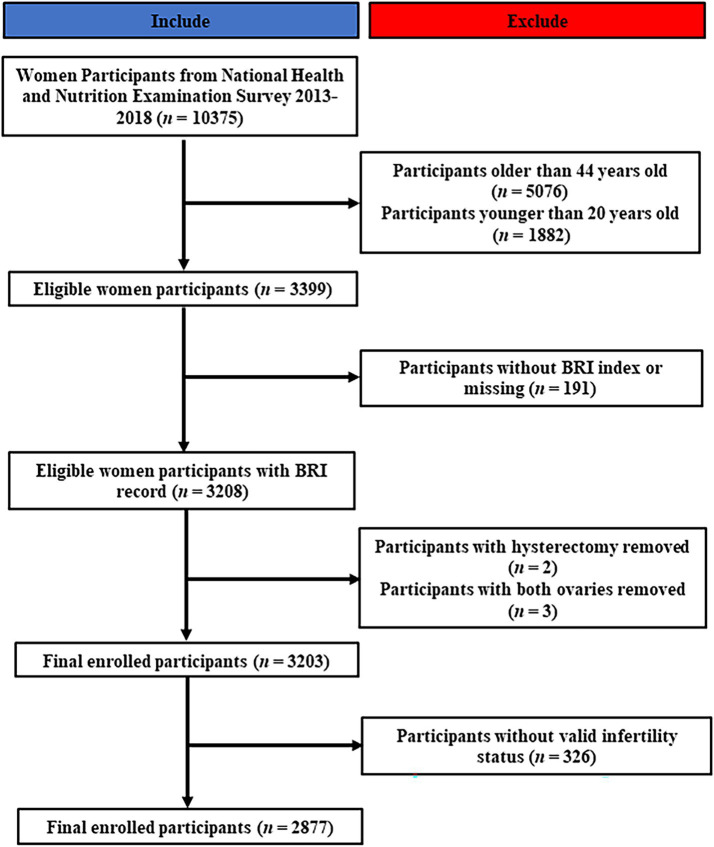
Flowchart of enrollment.

The NHANES study, a comprehensive cross-sectional investigation conducted on a national scale in the United States, is designed to assess the health and dietary patterns of the entire population, encompassing both adults and children (21, 22). This study has played a pivotal role in informing health policy decisions (23). Given the limited availability of infertility-related data from 2013 to 2018, our study initially focused on analyzing data from the 10,375 female participants in NHANES during this time period, applying rigorous inclusion and exclusion criteria. Initially, women who did not fall within the reproductive age range were excluded from the study, comprising 5,076 individuals over the age of 44 and 1882 individuals under the age of 20. Subsequently, participants with incomplete or absent BRI data (*n* = 191) were excluded, as well as those who had undergone hysterectomy or oophorectomy procedures (*N* = 5). Furthermore, 326 women were found to be lacking essential infertility-related data. Ultimately, a total of 2,877 participants were included in the study. The recruitment process is visually represented in Figure 1.

### BRI measurement

Anthropometric measurements, such as body height (BH), body weight, and waist circumference (WC), were collected by trained examiners at a mobile examination center equipped with standardized tools. Participants’ body mass was evaluated using calibrated platform scales with a precision of 0.1 kg, and their height was measured with stadiometers while standing, accurate to 0.1 cm. These measurements were taken with participants wearing light clothing and no shoes. Consistent with prior research, BRI was calculated using the formula developed by Thomas et al. (20):


$$BRI=364.2-365.5\times \sqrt{1-\frac{{\left(\frac{WC}{2\pi }\right)}^{2}}{{\left(0.5\times BH\right)}^{2}}}$$

### Self-reported infertility

Consistent with previous research, infertility is defined as the failure to achieve pregnancy after 1 year of unprotected intercourse, as self-reported by individuals (24–26). The presence of infertility was determined through responses to a questionnaire, with women indicating a positive response to either of two questions: “Have you attempted to conceive for at least 1 year without success?” or “Have you sought medical assistance for infertility?” being classified as ever infertile.

### Covariates

Demographic information, including age, gender, race/ethnicity, educational level, marital status, household income, smoking, and alcohol consumption habits, was collected through standardized questionnaires and in-person interviews. Furthermore, data on menstrual regularity, pelvic infections, use of female hormones, and contraceptive pill usage was obtained through face-to-face interviews. Ethnicity was classified into five distinct categories: non-Hispanic White, non-Hispanic Black, other Hispanic, Mexican American, and other racial groups. Academic achievement was categorized into three tiers: below high school, high school, and above high school. The BMI, calculated by dividing weight in kilograms (kg) by height in meters squared (m^2^), was utilized as the principal measure for assessing overweight and obesity, with BMI values surpassing 25 kg/m^2^ and 30 kg/m^2^ denoting overweight and obesity, respectively.

### Statistical methods

The NHANES study employed a sophisticated weighted sampling technique to collect data, requiring the weighting of all analyses to ensure the sample’s representativeness. Data was presented using percentages and 95% confidence intervals. T-tests and chi-square tests were applied in the baseline characteristics table to compare variables across different groups. The BRI variable was analyzed both as a continuous variable and divided into four groups for further statistical explanations. Weighted and unweighted multivariable logistic regression analyses were conducted to consider covariates related to infertility. Additionally, the RCS method was used to investigate the relationship between BRI and infertility. Subgroup analyses were performed to explore how BRI and infertility are linked in diverse populations. Statistical analyses were carried out using R software version 4.1.6, with statistical significance set at a two-tailed *p*-value <0.05.

## Results

### Demographical and clinical characteristics of the study population

In this study, strict criteria for participant selection were applied, including of 2,777 eligible female participants from the NHANES. Among the cohort, 63.7% were females aged 20–34, while 36.3% were females aged 35–44. Of the total participants, 358 were identified as having infertility conditions, with this subgroup generally being older than those without infertility. Analysis of baseline data highlighted differences in marital status and family income between the two groups. Specifically, women with infertility had higher family incomes, higher rates of obesity based on BMI measurements, irregular menstrual periods, and a higher prevalence of pelvic infections. They also reported more frequent past use of female hormones and birth control pills. No significant differences were found in race, education level, smoking and alcohol consumption between the groups, as indicated in Table 1. Furthermore, participants were divided into four groups based on BRI, with detailed comparisons of baseline demographic and clinical characteristics provided in Supplementary Table S1.

**Table 1:** Demographical characteristics of the study population.

	Overall (*n* = 2,777)	Non-infertility (*n* = 2,419)	Infertility (*n* = 358)	*p* value
Age, years				<0.0001
20–34 years	63.65 [58.66, 68.64]	65.72 [63.01, 68.42]	49.75 [42.41, 57.10]	
35–44 years	36.35 [32.29, 40.41]	34.28 [31.58, 36.99]	50.25 [42.90, 57.59]	
Race/ethnicity				0.2
White	55.86 [48.28, 63.44]	55.11 [50.33, 59.90]	60.87 [53.86, 67.87]	
Black	13.29 [10.82, 15.76]	13.36 [10.59, 16.12]	12.86 [9.38, 16.34]	
Mexican	11.93 [9.01, 14.84]	11.99 [8.98, 14.99]	11.53 [7.07, 15.98]	
Other Hispanic	8.07 [6.39, 9.75]	8.42 [6.73, 10.10]	5.75 [3.10, 8.39]	
Others	10.85 [9.26, 12.44]	11.13 [9.36, 12.90]	9.00 [5.98, 12.02]	
Education levels				0.32
Less than high school	3.25 [2.36, 4.13]	3.39 [2.41, 4.37]	2.30 [0.84, 3.76]	
High school or equivalent	28.14 [24.86, 31.41]	27.71 [24.49, 30.93]	31.00 [24.69, 37.31]	
College or above	68.62 [61.69, 75.55]	68.90 [65.32, 72.49]	66.70 [60.08, 73.33]	
Marital status, *n* (%)				<0.001***
Divorced	6.15 [4.91, 7.40]	6.26 [4.88, 7.64]	5.42 [3.19, 7.64]	
Living with partner	14.68 [12.58, 16.78]	15.20 [13.50, 16.90]	11.15 [7.21, 15.10]	
Married	44.06 [39.52, 48.60]	40.93 [38.06, 43.80]	65.06 [58.72, 71.40]	
Never married	31.71 [28.54, 34.88]	34.24 [31.66, 36.82]	14.71 [11.39, 18.02]	
Separated	3.15 [2.47, 3.84]	3.10 [2.39, 3.82]	3.50 [1.43, 5.56]	
Widowed	0.24 [0.06, 0.43]	0.26 [0.05, 0.46]	0.17 [−0.16, 0.49]	
Family income				0.03*
< 2000$	17.78 [15.66, 19.90]	19.11 [16.99, 21.23]	14.36 [10.58, 18.14]	
≥ 2000$	78.40 [71.93, 84.88]	80.89 [78.77, 83.01]	85.64 [81.86, 89.42]	
BMI, kg/m^2^				0.002**
Normal weight	36.58 [32.09, 41.07]	37.71 [34.68, 40.74]	29.45 [23.15, 35.75]	
Over weight	24.18 [21.79, 26.56]	25.03 [23.02, 27.04]	18.76 [13.27, 24.25]	
Obesity	39.09 [36.14, 42.03]	37.26 [34.82, 39.70]	51.79 [43.79, 59.80]	
Regular menstrual periods, (%)	90.09 [83.47, 96.70]	90.66 [89.18, 92.14]	86.24 [81.50, 90.97]	0.05*
Pelvic infection, (%)	4.67 [3.54, 5.79]	4.11 [3.05, 5.16]	8.56 [5.39, 11.74]	<0.001***
Female hormones taken, %	4.20 [2.97, 5.42]	3.53 [2.44, 4.63]	8.69 [3.95, 13.44]	0.01*
Birth control pills taken, %	72.72 [66.24, 79.20]	71.89 [69.29, 74.49]	78.64 [73.35, 83.93]	0.03*
Smoking, %	19.93 [17.32, 22.54]	19.70 [17.64, 21.76]	21.55 [15.91, 27.20]	0.49
Drinking, %	83.89 [77.40, 90.38]	86.31 [84.00, 88.61]	89.68 [84.44, 94.93]	0.2

Variables are presented as the proportion and 95% confidence interval. BMI, body mass index. ***p value < 0.001, **p value < 0.01, *p value < 0.05.

### Associations between BRI and prevalence of infertility

**Figure 2:**
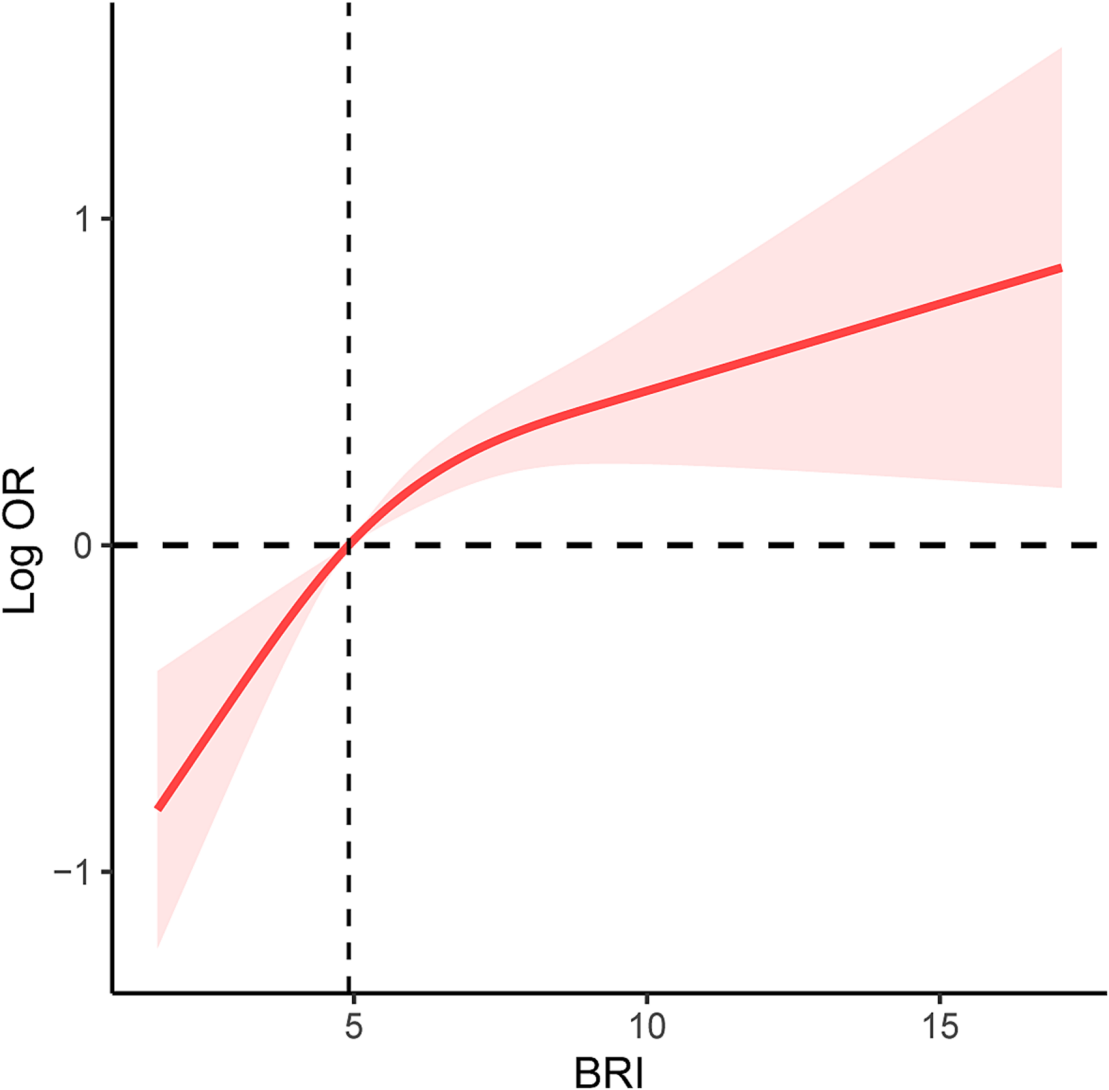
RCS curve of the association between BRI and prevalence of infertility among all participants. It was adjusted for age, marital status and race/ethnicity, education levels, family income, BMI, regular menstrual periods, pelvic infection, female hormones taken, birth control pills taken, drinking history and smoking history. RCS, restricted cubic spline; BRI, body round index; BMI, body mass index; OR, odds ratio.

A comprehensive weighted multivariable logistic regression analysis was conducted to investigate the relationship between BRI and infertility prevalence. Covariates such as age, marital status, ethnicity, education level, income, BMI, menstrual regularity, pelvic infection, hormone and birth control pill usage, alcohol consumption, and smoking history were adjusted to account for potential confounding factors. The results indicated a statistically significant relationship between BRI and infertility, regardless of whether BRI was treated as a continuous or categorical variable. In the fully adjusted model, the odds ratio (OR) for BRI as a continuous variable was 1.12 (95%CI: 1.05–1.19). When BRI was treated as a categorical variable with Q1 as the reference, the ORs were as follows: Q2 (OR: 1.82; 95%CI: 1.06–3.13), Q3 (OR: 2.05; 95%CI: 1.26–3.33), Q4 (OR: 2.94; 95%CI: 1.70–5.08; Table 2). While weighting methods may improve sample representativeness, some studies suggest that weighted and unweighted results can vary. In this study, an unweighted multivariable regression analysis was performed to examine the association between BRI and infertility. The findings of the unweighted analysis were found to align with those of the weighted analysis (Table 3). Furthermore, the RCS method was utilized to explore the correlation between BRI and infertility, revealing a notable linear rise in infertility rates with increasing BRI (Figure 2).

**Table 2:** Weighted multivariate logistic regression of the association between BRI and infertility.

	Non-adjusted model		Model I		Model II	
	OR [95% CI]	*p* value	OR [95% CI]	*p* value	OR [95% CI]	*p* value
Continuous BRI	1.12 [1.07,1.19]	<0.001***	1.11 [1.05,1.17]	<0.001***	1.12 [1.05,1.19]	<0.001***
BRI -Q1	Reference	-	Reference	-	Reference	-
BRI -Q2	1.70 [1.03, 2.80]	0.01*	1.70 [1.03, 2.80]	0.04*	1.82 [1.06, 3.13]	0.03*
BRI -Q3	1.88 [1.24, 2.83]	<0.001***	1.88 [1.24, 2.83]	0.004**	2.05 [1.26, 3.33]	0.01*
BRI -Q4	2.60 [1.59, 4.24]	<0.001***	2.60 [1.59, 4.24]	<0.001***	2.94 [1.70, 5.08]	<0.001***

Data are presented as OR [95% confidence interval]. Model I adjusted for age, marital status and race/ethnicity. Model II adjusted for age, marital status and race/ethnicity, education levels, family income, BMI, regular menstrual periods, pelvic infection, female hormones taken, birth control pills taken, drinking history and smoking history. BRI, body round index. ***p value < 0.001, **p value < 0.01, *p value < 0.05.

**Table 3:** Unweighted multivariate logistic regression of the association between BRI and infertility.

	Non-adjusted model		Model I		Model II	
	OR [95% CI]	*p* value	OR [95% CI]	*p* value	OR [95% CI]	*p* value
Continuous BRI	1.13 [1.09,1.17]	<0.001***	1.11 [1.07,1.16]	<0.001***	1.12 [1.07,1.16]	<0.001***
BRI -Q1	Reference	-	Reference	-	Reference	-
BRI -Q2	1.39 [0.97, 2.00]	0.07	1.30 [0.91, 1.88]	0.15*	1.37 [0.93, 2.02]	0.03*
BRI -Q3	1.70 [1.20, 2.42]	0.003**	1.56 [1.09, 2.24]	0.02*	1.71 [1.16, 2.52]	0.01*
BRI -Q4	2.62 [1.89, 3.67]	<0.001***	2.36 [1.68, 3.34]	<0.001***	2.54 [1.77, 3.70]	<0.001***

Data are presented as OR [95% confidence interval]. Model I adjusted for age, marital status and race/ethnicity. Model II adjusted for age, marital status and race/ethnicity, education levels, family income, BMI, regular menstrual periods, pelvic infection, female hormones taken, birth control pills taken, drinking history and smoking history. BRI, body round index. ***p value < 0.001, **p value < 0.01, *p value < 0.05.

### Subgroup analysis on the associations between BRI and prevalence of infertility

**Figure 3:**
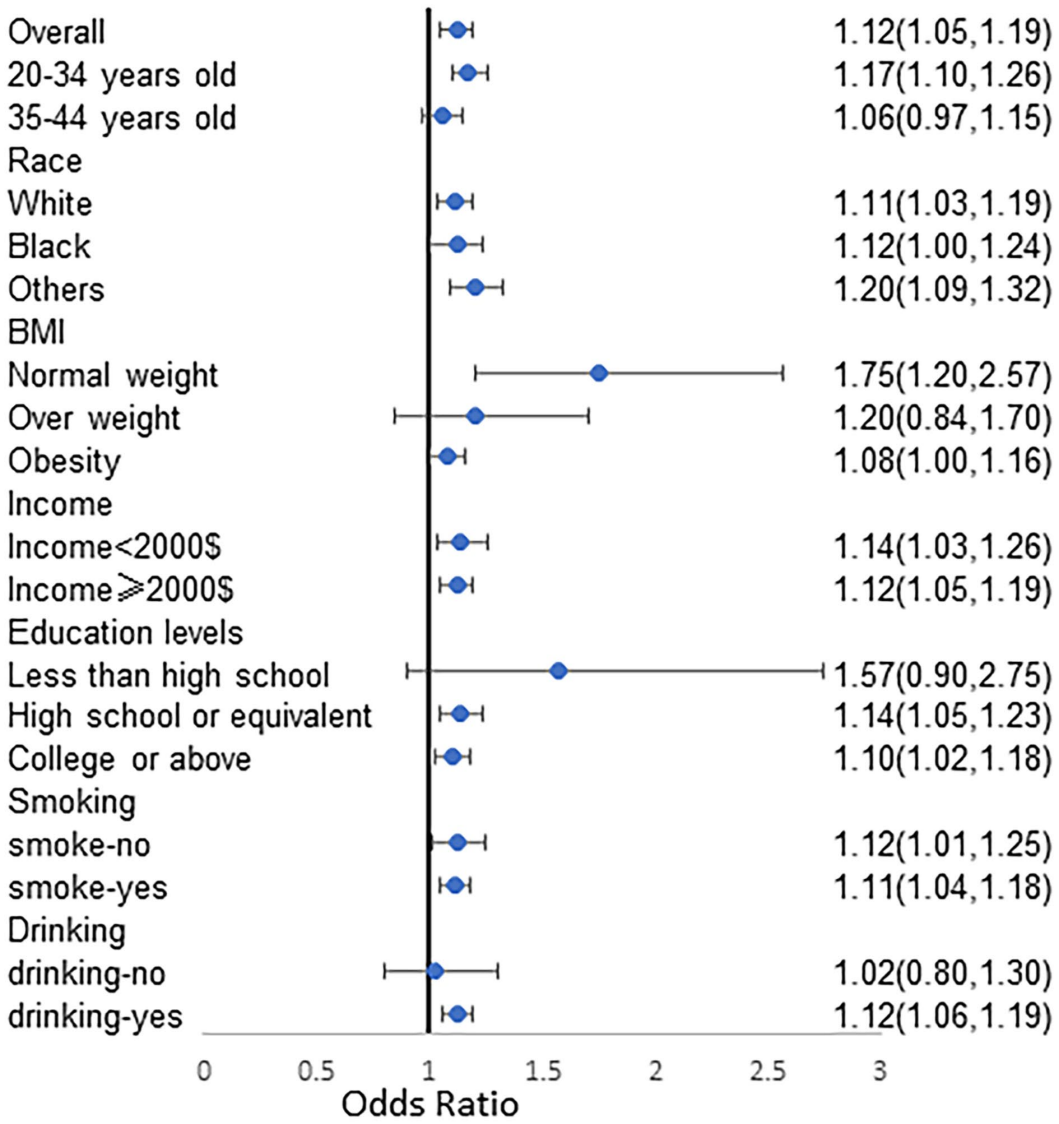
Subgroups analyzes stratified by age, race, family income, education levels, smoking and drinking for the association between BRI and prevalence of infertility. Analyses were adjusted for age, marital status and race/ethnicity, education levels, family income, BMI, regular menstrual periods, pelvic infection, female hormones taken, birth control pills taken, drinking history and smoking history. BRI, body round index; BMI, body mass index; OR, odds ratio.

**Figure 4:**
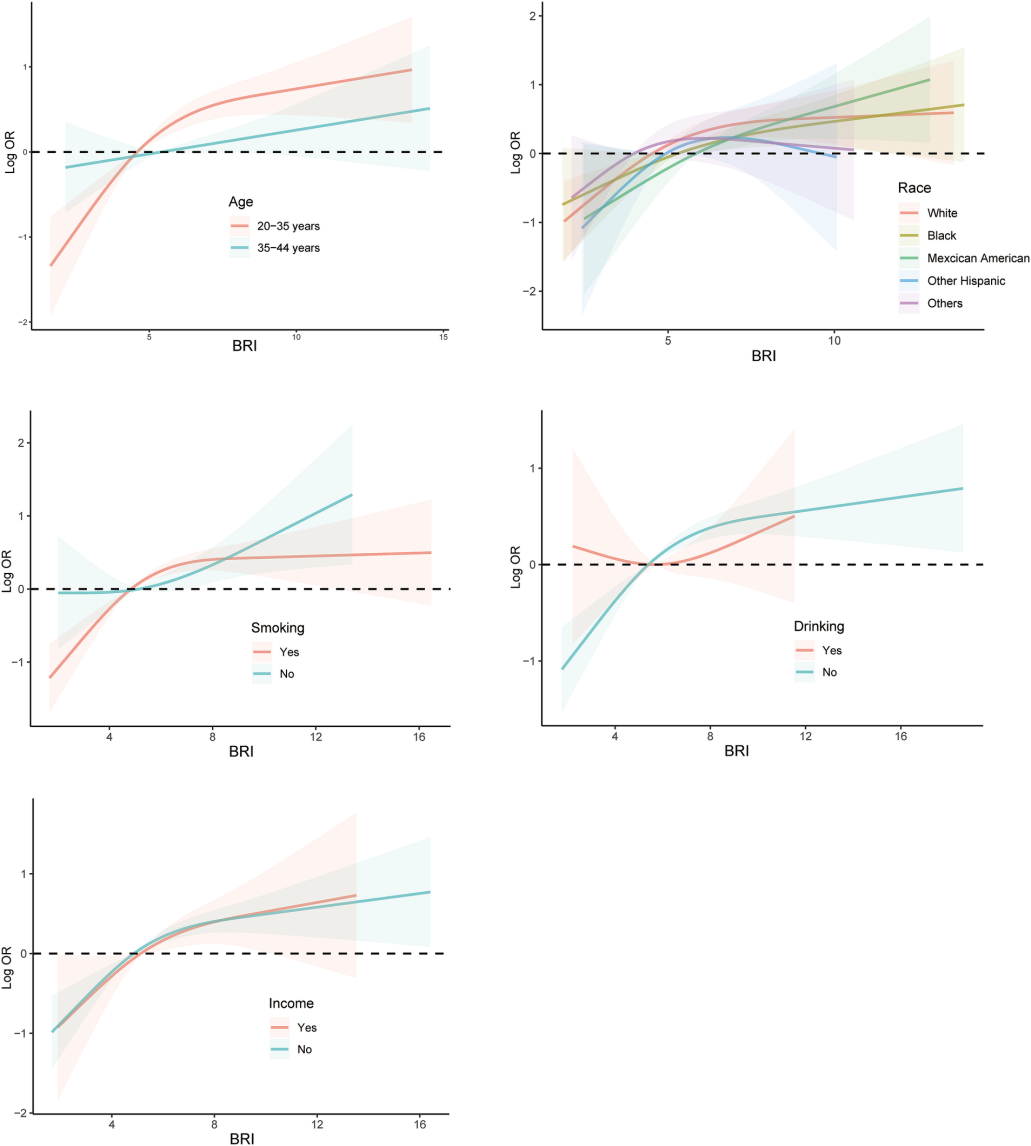
Subgroups RCS curves for the association between BRI and prevalence of infertility among populations with different characteristics. Analyses were stratified by age, race, smoking, drinking, and family income. RCS analyses were adjusted for age, marital status and race/ethnicity, education levels, family income, BMI, regular menstrual periods, pelvic infection, female hormones taken, birth control pills taken, drinking history and smoking history. RCS, restricted cubic spline; BRI, body round index; BMI, body mass index; OR, odds ratio.

In this study, detailed subgroup analyses to explore the relationship between BRI and infertility across various demographics. The findings revealed a positive correlation between BRI and infertility occurrence rates in several subgroups. However, this correlation did not reach statistical significance among older adult patients, overweight individuals, those with an educational level below high school, and non-drinkers. Conversely, statistically significant positive correlations between BRI and infertility were observed in other demographic groups, as illustrated in Figure 3. Furthermore, RCS spline analyses were performed on diverse populations characterized by varying ages, races, smoking and drinking habits, and income levels. The results indicated a significant positive correlation between BRI and infertility across a range of demographic variables. Specifically, as BRI levels increased, there was a consistent trend of rising infertility rates observed among different population subsets (Figure 4).

### Predictive value of BRI on the prevalence of infertility

**Figure 5:**
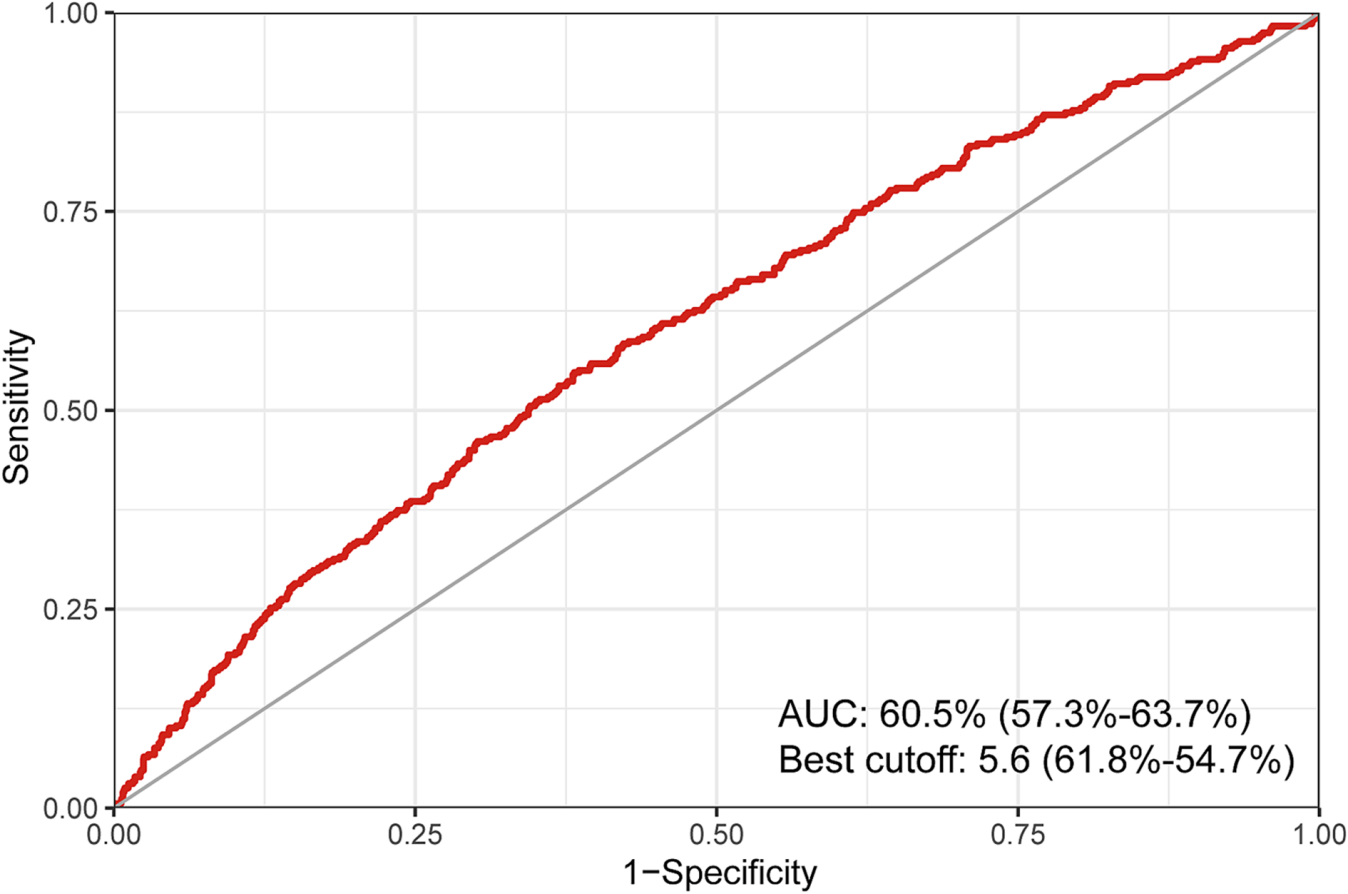
ROC curve of the predictive value for BRI on the prevalence of infertility. ROC, restricted cubic spline; BRI, body round index.

In addition, an examination was conducted to assess the predictive capacity of BRI in relation to infertility through the utilization of ROC curves. It was determined that BRI demonstrates a favorable predictive capability for fertility, as evidenced by an area under the curve (AUC) of 60.5% (57.3%—63.7%). The optimal cutoff value was identified as 5.6, yielding a sensitivity of 61.8% and a specificity of 63.7% (Figure 5).

## Discussion

In this cross-sectional study with a nationally representative sample, we investigated the relationship between BRI and infertility in women aged 20 to 45. Our findings reveal a notable link between BRI and the likelihood of infertility, even after accounting for potential confounding factors. BRI has the potential to serve as a predictive marker for infertility prevalence, which could inform strategies for prevention and treatment. Managing weight, decreasing abdominal circumference, and subsequently lowering BRI levels may potentially mitigate the prevalence of infertility.

This study is the first to directly investigate the relationship between the BRI and female infertility. Previous research has suggested that obesity can lead to hormonal imbalances and endocrine dysfunction, which may affect infertility (27, 28). However, the mechanism of increased BRI levels causing infertility prevalence in women remains unclear, and there exist several possible explanations. Excessive fat tissue, especially in the abdominal area, can raise estrogen levels in obese women, disrupting hormonal balance and causing changes in ovulation and menstrual cycles (29–33). Additionally, obesity can worsen ovulation problems, especially in cases of abdominal obesity, which are associated with hormonal imbalances such as PCOS (34). This form of obesity is also linked to insulin resistance and high blood sugar levels (35). Furthermore, inflammatory substances released by abdominal fat can negatively impact fertility and the receptivity of the endometrium to support a pregnancy. Moreover, obesity and excess fat can contribute to psychological issues like low self-esteem and anxiety in women, which can subsequently affect fertility (36).

BRI is a novel measurement index that offers advantages over the traditional BMI by specifically addressing the crucial health issue of abdominal obesity (37). Unlike BMI, which solely relies on height and weight, the BRI takes into account waist circumference, providing a more holistic perspective on body shape (38). This feature enhances the accuracy of the BRI in identifying abdominal obesity, particularly in individuals with excess abdominal fat, leading to a more precise evaluation of this condition (39). Moreover, the BRI is less influenced by muscle mass, making it a more dependable indicator of the relationship between fat content and health risks in individuals with higher muscle mass (40–43). Besides, the advantage of the BRI over WC is its incorporation of height information. This dual consideration enables the BRI to more comprehensively reflect an individual’s body shape characteristics and fat distribution. Consequently, the BRI is better suited for assessing diseases closely linked to abdominal obesity. Numerous studies have demonstrated that BRI is more strongly associated with the risk of metabolic disorders like diabetes and cardiovascular disease compared to BMI (13, 44). Furthermore, studies have shown that BRI can significantly determine the presence of insulin resistance (45). These results suggest that the BRI may offer a more precise reflection of the connection between body fat distribution and health risks (23).

Our study investigated the association between female infertility and BRI, revealing a positive correlation between BRI and a higher prevalence of infertility in both unadjusted and adjusted models. It is widely acknowledged that BRI serves as an indicator for obesity and reproductive issues. Our findings were consistent with anticipated outcomes. However, the precise mechanism through which elevated BRI levels contribute to the prevalence of female infertility remains unclear. Potential explanations include disruptions in fatty acid metabolism in visceral adipose tissue due to obesity, leading to excessive accumulation of fatty acids in various tissues. This accumulation can result in insulin resistance, fatty acid peroxidation, hormonal imbalances, and ultimately impact ovulation and ovum quality. Moreover, visceral adipose tissue, being an active endocrine tissue, can generate inflammatory mediators such as tumor necrosis factor α and interleukin-6. The excessive release of these mediators may trigger a chronic inflammatory response, causing damage to vascular endothelial cells and reducing endometrial receptivity (46, 47). Should further evidence emerge, it would be beneficial for individuals to identify the optimal control range for managing body size.

This study has various strengths. Firstly, the research utilized data from the NHANES database, including all eligible participants available. With a large number of participants included, the conclusions drawn can be considered more reliable. Secondly, the NHANES database employs complex stratified sampling methods, and all data analyses in the study were conducted using weighted analysis, enhancing the representativeness of the findings. Thirdly, detailed stratified analyses in subgroups revealed significant relationships between BRI and infertility across different populations, further bolstering the reliability of the research. Finally, both weighted and unweighted analyses were employed to validate the conclusions, and the results from both analyses were consistent.

This study also has some limitations. Firstly, it was only a cross-sectional study, limiting the ability to infer causal relationships. Secondly, the self-reported nature of the outcome measure, infertility, may introduce reporting bias. Thirdly, the study was conducted solely within the United States, and while it included multiple ethnicities, the generalizability of the findings to other countries and regions requires further confirmation through large-scale prospective cohort studies.

## Conclusion

Participants in this study were recruited from NHANES, specifically targeting women of childbearing age. The study results indicated a significant positive correlation between BRI and the prevalence of infertility. As the BRI level increased, the prevalence of infertility also increased linearly. This correlation was consistent across demographic characteristics. Additionally, BRI may serve as a valuable predictor of infertility prevalence. In order to improve fertility, it is recommended that women prioritize the maintenance of a balanced diet, regular physical activity, and the management of a healthy weight and waist circumference. Should further research corroborate these conclusions, it would be advantageous for individuals to ascertain the optimal BRI for the regulation of body size. Future prospective cohort studies should delve into the association and elucidate the underlying mechanisms.

## Data Availability

The original contributions presented in the study are included in the article/Supplementary material, further inquiries can be directed to the corresponding authors.
