# Supplementary material for: Association of body roundness index with female infertility: 2013–2018 NHANES
Source: Front Nutr. 2024 Oct 30;11:1416637. doi: 10.3389/fnut.2024.1416637 (PMC11561710; doi:10.3389/fnut.2024.1416637)
Supplement: Supplementary file 1 [file Table_1.docx]

**Table S1. Demographical Characteristics of the Study Population.**

|  | **Overall (n = 2777)** | **BRI-Q1** | **BRI-Q2** | **BRI-Q3** | **BRI-Q4** | ***p* value** |
| --- | --- | --- | --- | --- | --- | --- |
| **Age, years** |  |  |  |  |  | <0.001*** |
| **20-34 years** | 63.65 [58.66, 68.64] | 75.26[70.82, 79.69] | 63.18[57.92, 68.44] | 57.51[52.73, 62.28] | 56.91[52.41, 61.41] |  |
| **35-44 years** | 36.35 [32.29, 40.41] | 24.74[20.31, 29.18] | 36.82[31.56, 42.08] | 42.49[37.72, 47.27] | 43.09[38.59, 47.59] |  |
| **Race/ethnicity** |  |  |  |  |  | <0.001*** |
| **White** | 55.86 [48.28, 63.44] | 63.71[58.27, 69.14] | 58.12[52.09, 64.15] | 47.72[41.61, 53.84] | 52.25[46.04, 58.46] |  |
| **Black** | 13.29 [10.82, 15.76] | 10.80[8.05, 13.55] | 10.51[7.94, 13.07] | 15.08[11.24, 18.92] | 17.43[13.14, 21.72] |  |
| **Mexican** | 11.93 [9.01, 14.84] | 5.06[3.02, 7.10] | 9.91[7.07, 12.75] | 17.83[13.47, 22.18] | 16.23[11.57, 20.89] |  |
| **Other Hispanic** | 8.07 [6.39, 9.75] | 7.44[5.38, 9.50] | 8.54[5.66, 11.41] | 9.01[6.22, 11.80] | 7.40[5.69, 9.10] |  |
| **Others** | 10.85 [9.26, 12.44] | 12.99[10.15, 15.83] | 12.93[10.04, 15.83] | 10.36[7.75, 12.97] | 6.69[4.95, 8.43] |  |
| **Education levels** |  |  |  |  |  | <0.001*** |
| **Less than high school** | 3.25 [2.36, 4.13] | 0.96[0.29, 1.63] | 3.19[1.70, 4.67] | 5.20[3.58, 6.82] | 4.02[2.35, 5.68] |  |
| **High school or equivalent** | 28.14 [24.86, 31.41] | 21.68[17.70, 25.66] | 24.36[19.63, 29.10] | 32.96[28.04, 37.88] | 34.94[30.73, 39.15] |  |
| **College or above** | 68.62 [61.69, 75.55] | 77.36[73.23, 81.49] | 72.45[67.56, 77.35] | 61.84[56.78, 66.90] | 61.05[56.21, 65.89] |  |
| **Marital status, *n* (%)** |  |  |  |  |  | <0.001*** |
| **Divorced** | 6.15 [4.91, 7.40] | 3.83[2.26, 5.40] | 7.05[4.48, 9.63] | 8.03[5.26, 10.81] | 6.01[4.22, 7.81] |  |
| **Living with partner** | 14.68 [12.58, 16.78] | 12.08[8.87, 15.29] | 15.23[11.94, 18.53] | 16.22[12.43, 20.02] | 15.55[12.56, 18.55] |  |
| **Married** | 44.06 [39.52, 48.60] | 38.31[33.90, 42.71] | 48.73[43.48, 53.98] | 45.61[39.70, 51.51] | 44.07[39.62, 48.51] |  |
| **Never married** | 31.71 [28.54, 34.88] | 44.40[39.67, 49.13] | 25.31[21.53, 29.09] | 25.74[21.10, 30.39] | 29.95[25.62, 34.28] |  |
| **Separated** | 3.15 [2.47, 3.84] | 1.31[0.52, 2.10] | 3.41[1.90, 4.92] | 3.81[2.48, 5.14] | 4.33[2.61, 6.05] |  |
| **Widowed** | 0.24 [0.06, 0.43] | 0.08[-0.08, 0.23] | 0.27[0.00, 0.54] | 0.58[-0.06, 1.22] | 0.08[-0.08, 0.25] |  |
| **Family income** |  |  |  |  |  | 0.09 |
| **< 2000$** | 17.78 [15.66, 19.90] | 19.92[16.95, 22.90] | 14.97[11.67, 18.28] | 18.77[14.83, 22.72] | 20.44[16.64, 24.24] |  |
| **≥ 2000$** | 78.40 [71.93, 84.88] | 80.08[77.10, 83.05] | 85.03[81.72, 88.33] | 81.23[77.28, 85.17] | 79.56[75.76, 83.36] |  |
| **BMI, kg/m^2^** |  |  |  |  |  | <0.001*** |
| **Normal weight** | 36.58 [32.09, 41.07] | 95.62[93.69, 97.55] | 39.65[35.45, 43.85] | 2.10[0.99, 3.21] | 0.00[0.00, 0.00] |  |
| **Over weight** | 24.18 [21.79, 26.56] | 4.38[2.45, 6.31] | 54.68[50.11, 59.25] | 36.62[32.58, 40.67] | 1.55[0.60, 2.50] |  |
| **Obesity** | 39.09 [36.14, 42.03] | 0.00[0.00, 0.00] | 5.67[3.33, 8.01] | 61.28[56.95, 65.61] | 98.45[97.50, 99.40] |  |
| **Regular menstrual periods, (%)** | 90.09 [83.47, 96.70] | 92.15[89.80, 94.51] | 90.29[87.05, 93.52] | 89.64[86.57, 92.71] | 87.95[84.61, 91.30] | 0.29 |
| **Pelvic infection, (%)** | 4.67 [3.54, 5.79] | 3.67[2.20, 5.14] | 3.56[2.07, 5.04] | 5.13[3.20, 7.05] | 6.64[3.87, 9.41] | 0.07 |
| **Female hormones taken, %** | 4.20 [2.97, 5.42] | 2.25[0.78, 3.72] | 5.22[3.04, 7.41] | 5.96[3.33, 8.60] | 3.62[1.88, 5.35] | 0.04* |
| **Birth control pills taken, %** | 72.72 [66.24, 79.20] | 72.82[69.00, 76.64] | 73.97[69.59, 78.34] | 72.51[69.30, 75.71] | 71.58[67.10, 76.05] | 0.83 |
| **Smoking, %** | 19.93 [17.32, 22.54] | 18.61[14.96, 22.26] | 18.71[15.98, 21.45] | 19.16[15.59, 22.73] | 23.54[19.37, 27.72] | 0.12 |
| **Drinking, %** | 83.89 [77.40, 90.38] | 90.68[87.46, 93.89] | 84.93[80.52, 89.34] | 83.80[80.37, 87.23] | 87.02[84.69, 89.34] | 0.01* |

Variables are presented as the proportion and 95% confidence interval. BMI, body mass index. *** *P* value<0.001, ** *P* value<0.01, * *P* value<0.05.
